# Challenges and Opportunities in Engineering the Electronic Structure of Single-Atom Catalysts

**DOI:** 10.1021/acscatal.2c05992

**Published:** 2023-02-14

**Authors:** Vera Giulimondi, Sharon Mitchell, Javier Pérez-Ramírez

**Affiliations:** Department of Chemistry and Applied Biosciences, Institute for Chemical and Bioengineering, ETH Zurich, Vladimir-Prelog-Weg 1, 8093 Zurich, Switzerland

## Abstract

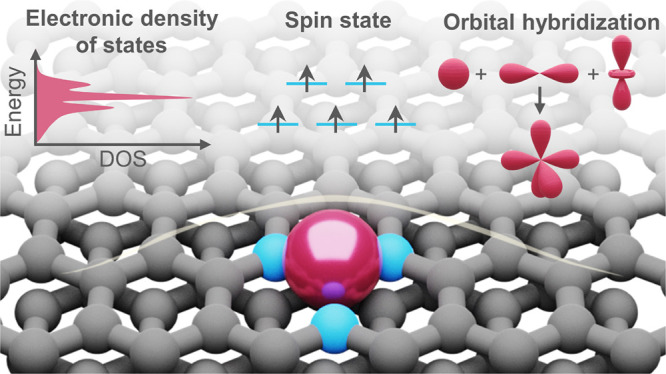

Controlling the electronic structure of transition-metal single-atom heterogeneous catalysts (SACs) is crucial to unlocking their full potential. The ability to do this with increasing precision offers a rational strategy to optimize processes associated with the adsorption and activation of reactive intermediates, charge transfer dynamics, and light absorption. While several methods have been proposed to alter the electronic characteristics of SACs, such as the oxidation state, band structure, orbital occupancy, and associated spin, the lack of a systematic approach to their application makes it difficult to control their effects. In this Perspective, we examine how the electronic configuration of SACs can be engineered for thermochemical, electrochemical, and photochemical applications, exploring the relationship with their activity, selectivity, and stability. We discuss synthetic and analytical challenges in controlling and discriminating the electronic structure of SACs and possible directions toward closing the gap between computational and experimental efforts. By bringing this topic to the center, we hope to stimulate research to understand, control, and exploit electronic effects in SACs and ultimately spur technological developments.

## Introduction

Single-atom heterogeneous catalysts (SACs), relative newcomers in the field of catalysis over supported transition metals, are driving a revolution, in terms of the application scope, the utilization of scarce metals, and atomically precise design.^1−4^ Compared to the extended metal surfaces found in nanoparticles, the spatial isolation of atoms on suitable carrier materials results in distinct geometric and interdependent electronic properties.^5^ Rather than having a mixture of sites (e.g., at different faces, edges, vertices, defects, or interfaces) as present in clusters or nanoparticles, all metal atoms in SACs directly interact with surface sites. Consequently, the properties of support surfaces (e.g., crystalline structures, presence of defects, lattice strain, surface area) are under increasing scrutiny as they now play a much more critical role in defining the coordination environment and uniformity of metal species.^6−8^ The downsizing of metals to single atoms often results in unique electronic structures with multiple discrete and separated states, compared to the continuous bands characteristic of their bulk counterparts. These changes have stimulated great interest in developing approaches for modulating the electronic structure by controlling the composition and nanostructure of SACs.^9,10^

Understanding the effect of the electronic structure of SACs on their interaction with atoms and molecules is fundamental for elucidating catalytic reaction mechanisms and optimizing their design. For transition-metal-based SACs, theoretical studies have demonstrated the crucial role of charge transfer and withdrawal from *d*-orbitals in governing catalytic performance.^11,12^ The development of several general relationships has helped experimentalists and theoreticians to rationalize the catalytic behavior of bulk transition metals, arguably the most successful of which is the well-known *d*-band model. Nonetheless, as highlighted by several recent works,^5,13−16^ the performance of SACs can significantly differ from that of extended metal surfaces, calling for a reanalysis of the suitability of existing general models, e.g., to account for local charges or spin polarization. Beyond heterogeneous catalysts, researchers frequently highlight the resemblance of SACs to organometallic complexes and have explored descriptors such as molecular orbitals and oxidation states.^17,18^ However, it has also been shown that the coordination of metal atoms to solid supports can differ in nature, compared to ligands,^18,19^ and the impacts are not yet fully understood.

Multiple excellent reviews already provide a comprehensive picture of approaches to stabilize metal atoms on suitable carriers and applications where they exhibit promising performance.^3,13,20−24^ Recently, there has been considerable progress in discriminating diverse electronic properties of SACs and understanding their potential effects in catalysis.^8,13,25^ Nevertheless, these are assessed on a case-by-case basis, calling for the development of a unified approach to systematically investigate all electronic features of the active ensembles. This Perspective aims to highlight the state-of-the-art in engineering the electronic structure of SACs, analyzing the property changes observed compared to extended metal surfaces, the approaches pursued to modulate and characterize them, and their impact on the mechanisms of thermocatalytic, electrocatalytic, and photocatalytic processes. By highlighting the current opportunities and challenges, we hope to guide research and inspire future contributions in this exciting area.

## Electronic Properties of SACs

Researchers often describe the electronic characteristics of single-atom catalysts as unique relative to extended transition-metal surfaces.^1^ This tendency likely derives from the legacy of downsizing supported metal nanoparticles toward improving their utilization in catalytic applications where the reference was typically the bulk metal. However, the distinct properties are not a unique feature so much as a direct consequence of the fact that a single atom exhibits no periodicity and, therefore, no *d*-band as such if there is no strong interaction with the support. Early studies focused on differences in the oxidation state experimentally detected by X-ray photoelectron spectroscopy (XPS) and X-ray absorption spectroscopy (XAS), which were linked to charge transfer between the isolated metal atoms and the support is consistent with density functional theory predictions. In contrast to the zerovalent character associated with bulk metals, single-atom sites frequently exhibit positive or, less commonly, negative charge states, which depend intimately on their coordination environments (e.g., if they occupy cation or anion vacancies).^26^ The possibility to stabilize metal cations is attractive for several applications, especially those requiring oxidation capacity. However, high-valent metals are not suitable for all applications, and to preserve the benefits of enhanced metal utilization associated with high dispersion, interest in controlling the oxidation state of SACs and developing zerovalent systems has rapidly grown.

### Electronic Metal Support Interactions

Understanding how metal atoms interact with support materials and other exchangeable ligands is crucial for designing and optimizing the electronic structure of SACs. The term electronic metal–support interaction (EMSI) is often used to describe the chemical bonding (van der Waals, covalent, or ionic) and charge transfer between isolated metal atoms and their support.^6,27,28^ The strength of this interaction can range from weak to strong, and can depend on factors such as the type of support, the coordination site of the metal, and the presence of adsorbed species.^29^ Recent research suggests that controlling EMSI by selecting metals with higher electronegativity than the surrounding atoms in the support can be used to further develop anionic SACs.^30^

### Orbital Hybridization

**Figure 1:**
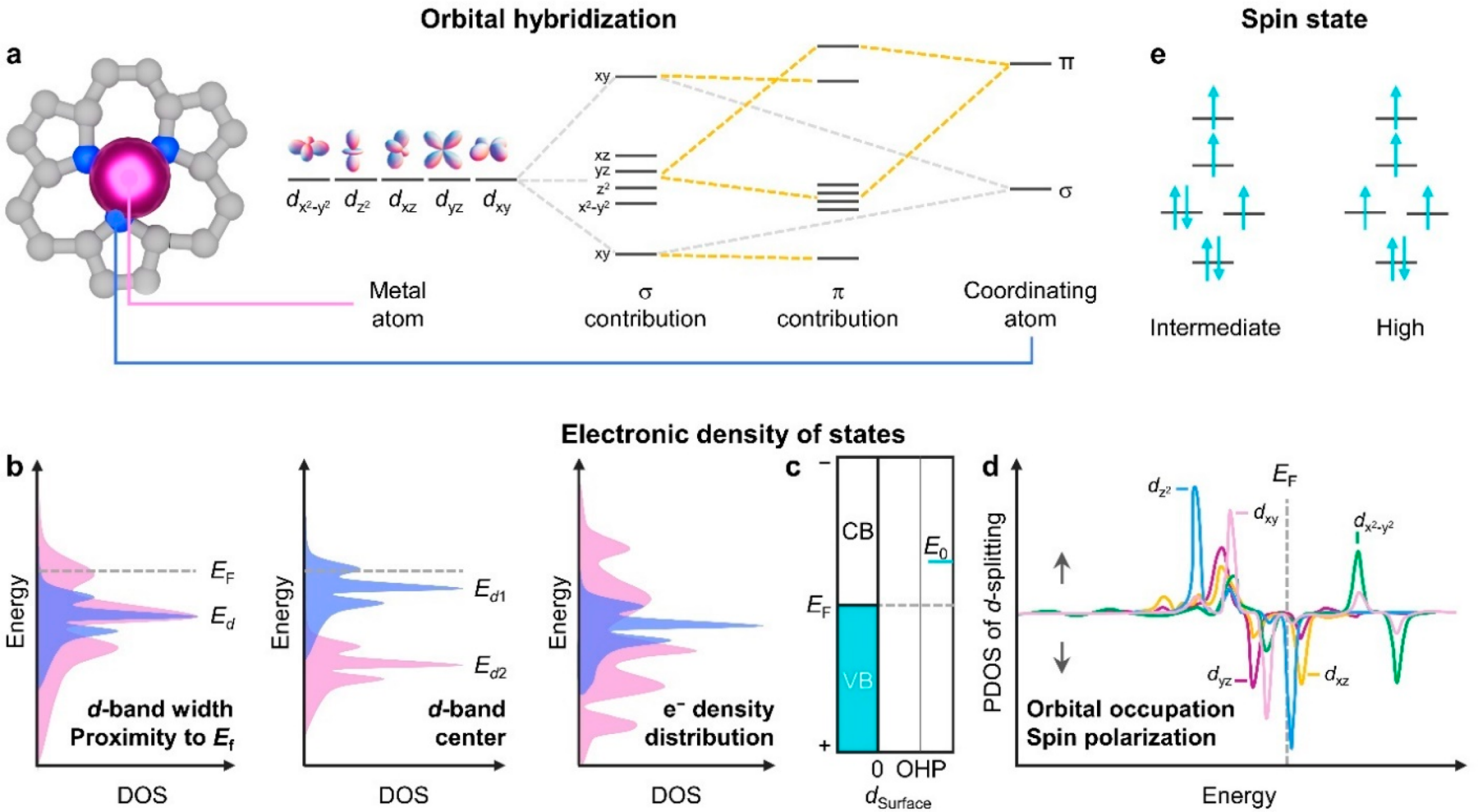
Schematic illustrations of key electronic characteristics of SACs. (a) Orbital hybridization, (b–d) electronic density of states, and (e) spin state. Abbreviations: (P)DOS, (Projected) density of states; *E*_F_, energy of the Fermi level; *E*_*d*_, energy of the *d*-band center; *E*_0_, metal reduction potential; *d*_surface_, distance from surface; OHP, outer Helmholtz plane; VB, valence band; and CB, conduction band. [Panel (c) was adapted with permission from ref (48). Copyright 2019, AAAS.]

Describing EMSIs and their effects on the electronic structure more rigorously requires the consideration of orbital diagrams and potential interactions (Figure 1a). Conceptually, the metal–insulator transition from extended band to molecular orbital structures upon decreasing the particle size to single atoms is well-known.^31^ Controlling orbital hybridization by varying the chemical identity and geometry of the metal center and coordinating atoms and their associated orbital energies and overlap is increasingly pursued as a strategy to optimize SAC performance, e.g., as illustrated in the design of water electrolysis technologies.^32−35^ Orbital hybridization can only occur when the orbitals of the support have similar energies to those of the metal center. Because of the prevalent role of *p*-block elements as coordinating atoms in SAC supports, *d*-*sp* orbital hybridization dominates the electron structure of most reported transition-metal SACs.^36,37^ Obviously, the criterion of orbital energy matching may be met to a greater or lesser extent.^38^ In the design of single-atom alloys (SAAs), weak wave function mixing between minority and majority elements limits the *d*–*d* hybridization, resulting in free-atom-like atomic states on the minority element.^16,39^ SACs with electronic structures resembling free atoms have also been reported to form under reaction conditions due to weakening of the metal–support interaction, as observed for Pt_1_/NC in the hydrogen evolution reaction (HER).^40^

The importance of orbital hybridization in regulating the *d*-band structure of SACs is increasingly recognized and presents numerous opportunities for tuning the interaction with reactants and intermediates.^41^ Studies analyzing the orbitals, closest to the Fermi level of SACs, are growing and indicate that the spatial structures of frontier *d*-orbitals play a vital role in determining the chemical and catalytic activities of SACs.^38^ Besides the coordinating first nearest-neighbor atoms, electron transfer may occur from second neighbors or beyond.^36^ Future efforts to gain a precise understanding of the effects of the extended structure of SAC sites will help establish the potential for tailoring the performance.

### *d*-Band Structure

The action of transition metals to facilitate the desired creation or cleavage of chemical bonds stems from the ability to provide unpaired *d*-electrons or empty *d*-orbitals to adsorb intermediates during the catalytic cycle, thereby requiring a comprehensive description of the *d*-band structure. One of the most widely used approaches to link catalytic activity to the electronic structure of transition-metal surfaces is the *d*-band model,^42^ which provides an approximate description of bond formation. In SACs, it is valuable to describe the interaction of the *d*- and, in some cases, *s*- or *p*-states of metal centers with the valence states of surrounding atoms in the support. The average energy of the *d*-electronic state of a metal center, described by the *d*-band center, width, and position of the upper edge relative to the Fermi level, have all been considered as structural descriptors for guiding the design of SACs (Figure 1b). Several works have demonstrated the possibility of tuning the *d*-band center, an indicator of the extent of *d*-band filling, and *d*-bandwidth by engineering the interaction of single atoms with support materials.^43−45^ Compared to extended metal surfaces, a downshift in the *d*-band center of single atoms is often observed due to electron transfer to the support, and the *d*-bandwidth is typically narrower. The narrowing of the *d* band originates from the loss of periodicity, which implies that the band structure of SACs can only originate from the hybridization with the electronic states of the support. Broader *d*-band widths in SAAs have been linked to more pronounced spin–orbital splitting of 5*d* compared to 3*d* and 4*d* metals.^25^

The strong dependence of the electronic states in SACs on the interaction with the support also permits regulation of the energy of the Fermi level, which unsurprisingly often differs from the bulk metal.^46^ For example, the incorporation of Cu atoms in a Ag lattice was predicted and subsequently experimentally demonstrated to reduce the Fermi level of Cu compared to the bulk metallic state.^47^ This presents interesting opportunities since the proximity of the electronic state of frontier orbitals to the Fermi level plays a crucial role in the bonding interaction with reaction intermediates, determining the gap between the highest occupied molecular orbitals (HOMOs) and lowest unoccupied molecular orbitals (LUMOs). In terms of SAC stability, the relative position of the Fe^3+^/Fe^2+^ reduction potential, compared to the Fermi level, was proposed to be critical to stabilizing active Fe^3+^ species on nitrogen-doped carbons (Figure 1c).^48^ Recent computational studies have stressed the importance of incorporating the ground and low-lying potential energy surfaces to properly account for the electronic structure of SACs. In some systems, such as Pt_1_/CeO_2_, the oxidation state of the metal may change due to dynamic charge transfer.^49^

Efforts to deepen structure–function relationships increasingly show the importance of considering the distinct spatial symmetry and energy levels of individual *d*-orbitals (Figure 1d).^50,51^ When isolated in 2D planes, the projected orbitals may interact with reactive intermediates to differing degrees, where in-plane projected *d*-orbitals may be completely inert, bringing into question the accuracy of using the properties of the total *d*-band as catalytic descriptors. Recent studies have also revealed the connection between the spin polarization of transition metals, charge transfer, and orbital interactions with reactive species.^52,53^ Since these effects are derived from the partial occupation of *d*-orbitals, this also calls for a more-detailed understanding of their individual properties.

### Spin State

When considering orbital filling, several different spin states may be energetically accessible and potentially coexist, depending on the oxidation state of the metal and the effects of orbital hybridization (Figure 1e), impacting the structure, reactivity, and magnetic properties of SACs.^54^ For example, the FeN_4_ moiety was reported to exhibit different electron configurations, including low (*d*_*xy*_^2^ *d*_*yz*_^2^ *d*_*xz*_^1^ *d*_z^2^_^1^), intermediate (*d*_*xy*_^2^ *d*_*yz*_^1^ *d*_*xz*_^1^ *d*_z^2^_^1^), and high (*d*_*xy*_^1^ *d*_*yz*_^1^ *d*_*xz*_^1^ *d*_z^2^_^1^ *d*_x^2^-y^2^_^1^) spin states. Spin state and transitions offer exciting opportunities, e.g., to accelerate and impose selectivity on electron transfer.^55,56^ More detailed examination of the changes in the density of states has also evidenced a redistribution in single atoms compared to bulk metal counterparts, resulting in unoccupied orbitals of a certain directional character near the Fermi level.^57^ Several spin-polarized SACs have been reported, e.g., Mn_1_@C_2_N, and the effect has been linked to enhanced adsorption and activation of certain molecules.^55^ Nevertheless, limitations in manipulating spin and its unambiguous assignment still impose challenges for understanding and exploiting the full scope of engineering local spin states, and further advances in the range of accessible spin states in SACs are expected. Future endeavors are encouraged not only to explore and discriminate the diversity of SAC electronic properties but also to develop synthetic strategies enabling control over them. The acquired knowledge will offer exciting potential to finely tune the metal site reactivity.

## Engineering Strategies

**Figure 2:**
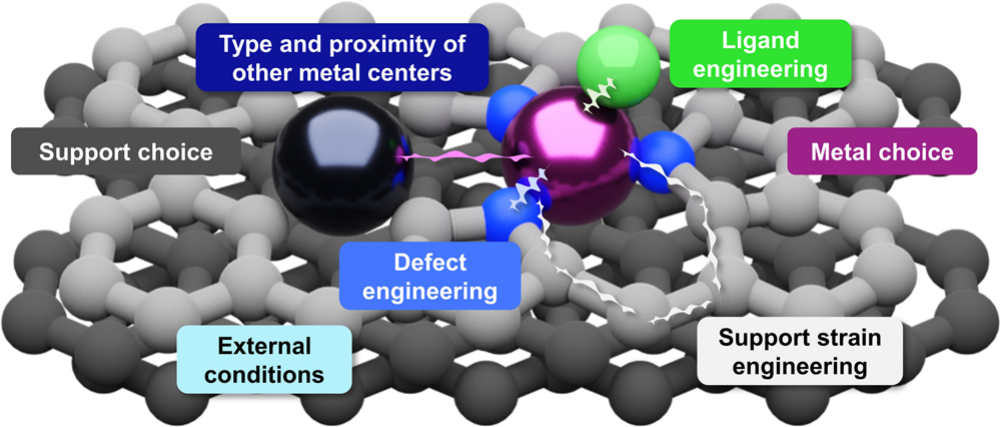
Overview of synthetic and operational strategies to engineer the electronic structure of SACs.

As with any other supported metal heterogeneous catalyst, the design of SACs begins with choosing suitable metal-host combinations for the targeted application. As opposed to catalysts featuring extended metal surfaces, the electronic and thus catalytic properties of the metal sites in SACs are tunable with atomic-scale rigor by controlling both the chemical identity and geometry of the coordinating atoms. This includes accounting for the interaction with (i) the anchoring sites in the host, (ii) any exchangeable ligands, and (iii) other metal atoms or defects that may be present in close proximity. The engineering of these properties together with attentive modulation of external factors, such as electromagnetic fields or applied voltage, provide immense potential for tailoring the electronic structure of SACs (Figure 2).^13^ Nevertheless, despite rapidly growing interest, our ability to exploit these opportunities for designing more effective catalysts remains primitive.

### Defect Engineering

Since the very successful formation of SACs requires stabilizing metal centers on support materials, research efforts have been extensively focused on host engineering. Defect sites in the host (e.g., vacancies, adatoms, heteroatoms) can trap single metal atoms by either covalent or ionic bonding in EMSI.^58^ While considerable progress has been made in tuning the catalytic performance via defect engineering, accurate evaluation of its impact on the metal center orbital configuration is still thwarted by intrinsic limitations of characterization and computational techniques (vide infra). Still, this information is central to unlocking the full scope of SAC electronic property design. Engineering of the host anchoring sites should enable the effective stabilization of the metal centers onto the support and adaptative bonding for optimized geometric and electronic properties, depending on the catalytic cycle in the targeted application.

**Figure 3:**
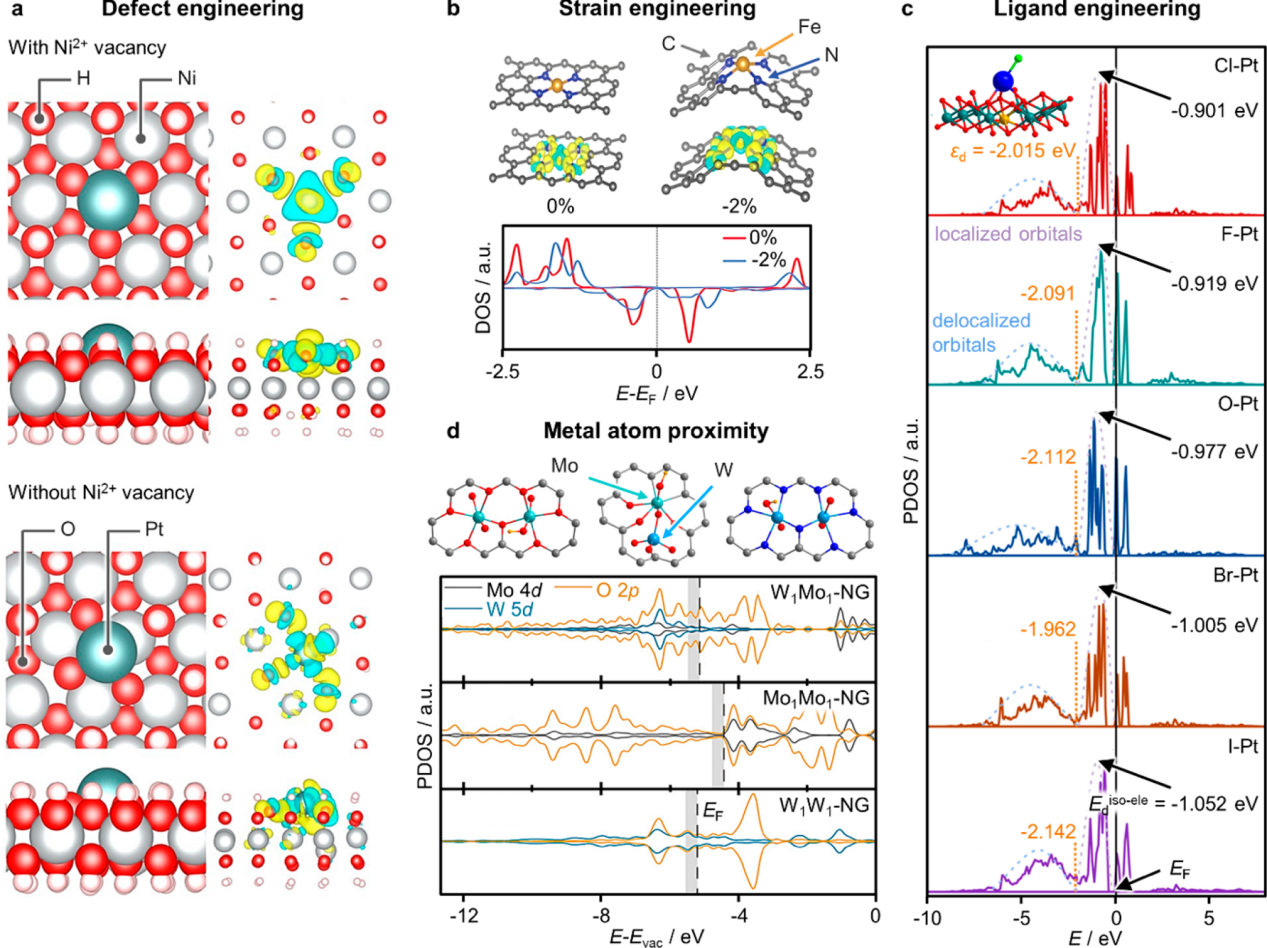
Examples illustrating how engineering strategies enable fine-tuning of SAC electronic properties. (a) Charge density distribution maps for a Pt atom adsorbed on a Ni(OH)_2_ support with or without Ni^2+^ vacancies. (b) Charge density distribution maps of N-doped carbon-supported Fe SACs in their fully relaxed and contracted geometries (0 and −2% applied strain), together with their corresponding DOS. (c) PDOS showing the impact of diverse ligands on the 5*d* band structures of Pt SACs supported on a NiFe-layered double hydroxide, evaluated based on the average energy levels of the Pt *5d* orbitals occupied by isolated electrons (*E*_d_^iso-ele^). (d) PDOS of metal (W and Mo) and O atoms for monometallic and bimetallic W–Mo dimers, supported on N-doped graphene (NG). [Panel (a) was adapted with permission from ref (59). Copyright 2018, Springer Nature. Panel (b) was adapted with permission from ref (72). Copyright 2019, John Wiley and Sons. Panel (c) was adapted with permission from ref (75). Copyright 2022, Springer Nature. Panel (d) was adapted with permission from ref (84). Copyright 2020, AAAS.]

Metal-based supports, spanning metal oxides, transition metals, metal carbides/nitrides (MXenes, where X = C or N), metal borides (MBenes), and transition-metal chalcogenides (containing Te, Se, or S anions), are attractive for SACs. In these systems, vacancy engineering has shown considerable promise to enhance the properties for various thermocatalytic and electrocatalytic applications. For example, generation of cationic Ni^2+^ vacancies in a nickel hydroxide support via a solvothermal procedure enhanced the stabilization of isolated Pt atoms over the support, reflecting greater charge transfer from the support to the Pt atoms and enhanced performance in diboration reactions (Figure 3a).^59^ Similarly, increasing the O-vacancy density in CeO_2_ led to the formation of abundant Ce^3+^ sites in place of Ce^4+^ ones, resulting in a shift in the bond between the Cu single atoms and the O atoms in the support from covalent to ionic.^58^ In addition to vacancy abundancy, applying tensile strain in Ru SACs, by modification of the curvature in the MoS_2_-nanotube support, successfully demonstrated how regulation of vacancy geometry could optimize not only the structure of the active site but also the orbital configuration.^60^ The DOS of Ru 3*d* orbitals shifted nearer the Fermi level, facilitating the activation of H_2_O molecules to generate intermediate H and OH species over the Ru single atoms in the HER. This effect, coupled with an improvement of the H_2_O adsorption properties of neighboring Mo atoms, and correspondingly facilitated local H_2_O mass transfer to active sites, gives rise to a synergistic effect between the Ru single atoms and host vacancies, which varies depending on the intensity of the applied strain. Besides *d*-band shifting, vacancy engineering can be a valuable approach to increase the density of states around the Fermi level, as reported for Pt single atoms confined in a double Mo–Ti MXene and selectively stabilized over Mo vacancies.^61^ The projected density of state (PDOS) revealed the promotion of electron transfer and, importantly, greater conductivity, boosting the SAC catalytic activity for the HER.

While the study of EMSI is common practice in heterogeneous catalysis, the single-site nature of SACs has prompted significant efforts into the atomic-level engineering of the metal center coordination environment for fine-tuning the active site electronic structure. Specifically, control over the type, number, and geometry of coordinating atoms in the host is an important strategy for regulating the *d*–*p* hybridization and related adsorption properties of the metal atoms (vide infra).^62,63^ Controlled arrangement of local atomic structures in the host is central to having suitable anchoring sites that covalently stabilize the isolated metal atoms on the support and form active sites with desirable electronic and catalytic properties.^64^ A prominent example is carbon-supported SACs, as various methods exist to introduce heteroatoms into carbon materials. N-functionalities have been commonly employed as anchoring sites, yielding M–N_*x*_C_*y*_ active sites. However, the strong electronegativity of the coordinating N atoms can result in high reaction Gibbs free-energy values for intermediate adsorption on the metal site, hampering the kinetics of catalytic reactions.^65−67^ To overcome this, a second heteroatom can be introduced in the metal center coordination environment and redistribute the electron density. This was achieved in Co SACs supported on a N-doped carbon by partially substituting coordinating N atoms with P, as confirmed by extended X-ray absorption fine structure (EXAFS). In this architecture, the coordinating P atoms promote charge depletion of the Co active sites, improving the energy barrier and free energies for the HER pathways on the Co sites.^68^

Similarly, tailoring the coordination number of Co atoms with the support enabled regulation of their electronic features. By simply controlling the pyrolysis temperature, species ranging from Co–N_4_ to Co–N_2_ were selectively generated on a zeolitic imidazolate framework (ZIF) carrier. Decreasing N-coordination led to an upward shift in the *d*-band center of the Co sites, resulting in stronger *CO_2_^–^ binding, while simultaneously promoting the desorption of the CO intermediate and, thus, superior selectivity toward CO.^69^ Nevertheless, variation of the coordination number with the support can also affect the active site geometric properties, which can result in asymmetries in the charge distribution within the active ensemble. This aspect was investigated in Fe SACs for shuttled lithium polysulfides, supported on two different N-rich carbons.^70^ One of these was derived by polymerization of pyrrole aiming to selectively yield Fe–N_3_C_2_ moieties, asymmetrically connected to three N and two C atoms, as opposed to a glucose-derived N-doped carbon that would form Fe–N_4_ moieties, symmetrically coordinated with four N atoms and commonly reported in the literature.^71−73^ Consistent with the asymmetric charge redistribution corroborated by Bader charge analysis, density functional theory (DFT) simulations predicted the position of the adsorbed S atoms not to be vertical to the Fe ones in the Fe–N_3_C_2_ moieties. This outcome suggested the activation of d_*x*^2^–*y*^2^_ and *d*_*xy*_ orbitals with the formation of additional π-bonds, elucidating (i) strengthened *d*–*p* orbital hybridization, (ii) facilitated interfacial electron transfer, and, thus, (iii) catalytically enhanced sulfur redox kinetics in the asymmetric Fe–N_3_C_2_ configuration, compared with the symmetric Fe–N_4_ one. Undoubtedly, these results prompt further studies on geometry-electronic property relations in SACs, whose establishment will require the development of characterization techniques able to resolve the differences in the electronic structure of active ensembles with distinct geometric architectures.

### Support Strain Engineering

An alternative strategy to regulate the active site geometry is the modification of the bond length between the metal centers and the coordinating atoms in the support, as reported in Ag SACs supported on a microporous hollandite manganese oxide for the oxidation of formaldehyde. Different interatomic distances in the two near-neighbor shells (Ag–O and Ag–Ag) were obtained simply by employing two distinct synthetic methods: anti-Ostwald ripening or wet impregnation. The stronger EMSI resulting in the former, reflected in upward-shifted *d*-orbital states of Ag, favors O_2_ activation by increasing the DOS of its antibonding π*-orbital and/or by depleting the π- and 5 σ-bonding orbitals.^28^ Although not explicitly stated, this phenomenon appears to be an example of applied local strain, dictated by the synthetic procedure. This concept was also investigated over ZIF-supported Fe SACs for the oxygen reduction reaction. FeN_4_ sites with contracted Fe–N bonds, obtained via harsh thermal treatment, exhibited lower activation energy to break the O–O bond. Analysis of the electronic structure by charge density maps shows how shortening of the Fe–N bonds affects the electronic structure, reflecting in a positive shift of the 3*d* orbitals of the Fe atom and greater charge transfer from the latter to the adjacent N atoms (Figure 3b).^72^

While all the presented strategies have proven successful in tailoring the orbital configuration on a case-by-case basis, the lack of systematic studies hinders the derivation of general guidelines for SAC electronic structure design via host engineering. To this end, future endeavors are encouraged to both critically evaluate the accuracy of the assessment of the engineering strategy impact on the metal center orbital configuration and explore the transferability of the proposed approach to other metal–support combinations. For these purposes, overcoming intrinsic limitations in currently available characterization techniques will also be crucial to allow experimental verification of predicted behaviors. To date, resolution of the oxidation and coordination environment mainly relies on bulk techniques (e.g., XAS). As opposed to crystalline hosts such as metals and metal oxides, the intrinsic inhomogeneity of amorphous carriers poses considerable challenges to the identification of active site structures.^74^ In carbon-based supports, this is further complicated by the similar scattering properties of the heteroatoms commonly employed as dopants (e.g., N). The development of novel techniques and analytical approaches is essential to resolve the type and abundance of diverse metal species populations and ultimately enable comparative studies among different metal–support systems.

### Ligand Engineering

Besides host engineering, the metal atom electronic features can be further modified by ligand engineering. Metal sites often present ligands, either remaining from the metal precursor employed in the synthetic procedure or generated under reaction conditions.^75^ Recently, a facile irradiation-impregnation procedure was developed to synthesize a range of Pt atoms supported on NiFe-layered-double-hydroxide with various axial ligands (−F, −Cl, −Br, −I, −OH). While experimental and computational analyses uncovered their role in determining the Pt atom valence state and *d*-orbital configuration (Figure 3c), the impact of axial ligand engineering was demonstrated by the tunable electrocatalytic activity of the Pt atoms for the HER. Another example is Fe SACs for thermocatalytic alkyne semihydrogenation, derived from a Fe(CO)_5_ precursor. Therein, CO ligands donate electron density to the Fe centers, as corroborated by both spectroscopic and computational analyses, improving their H_2_ adsorption properties.^76^ Similarly, the valence of Fe single atoms was reported to increase upon acquisition, during the oxygen reduction reaction, of OH^–^ ligands from the aqueous solution, rendering the adsorption of O_2_ more favorable.^77^ Acquiring a sound understanding of such self-adjusting mechanisms is central to exploring the full scope of ligand engineering strategies and optimizing catalytic performance.

### Type and Proximity of Other Metal Centers

The metal atom coordination sphere can be further engineered by integrating a second metal atom. For over a decade, alkali metals have been known to modify charge distribution in the transition-metal active site via electron density donation.^78,79^ Recently, a novel strategy that has raised considerable interest is the introduction of a second transition metal atom, often leading to unique catalytic synergies.^80−82^ When in either the first or second shell of the former metal atom, the presence of the second metal gives rise to electron density transfer between the two centers. Pioneering works on graphene-supported Pt–Ru and O-bridged Mo–W dimers for the HER demonstrated charge redistribution, leading to a lesser degree of occupation of the *d*-orbitals of the metal atoms and, consequently, to superior hydrogen adsorption properties.^83,84^ Interestingly, the latter study explored the differences in the electronic structures of bimetallic and monometallic dimers (W_1_Mo_1_–NG, Mo_1_Mo_1_–NG, and W_1_W_1_–NG, respectively; see Figure 3d). While in all three systems, the Mo, W, and O atoms exhibit unoccupied states, deriving from the covalent bonding between metal ions and O atoms, the bimetallic W_1_Mo_1_–NG exhibits an increased DOS for the occupied states from 0.30 eV to the Fermi level (*E*_F_), because of delocalized electrons. Another study based on machine learning proposed a distinct research direction, focusing on lanthanide–transition-metal combinations.^85^ To design superior electrocatalysts, *f*–*d* orbital interactions offer good prospects for a superior modulation of the *d*-band center with enhanced stability by less orbital repulsive forces than their *d*–*d* counterparts.

Interestingly, metal centers can also influence each other’s spin density. Evidence for this was observed in ferromagnetic Co SACs on metallic TaS_2_ monolayers for the oxygen evolution reaction (OER), exchanging interactions with neighboring Co sites incorporated in the support in place of Ta atoms. The latter Co species increase the spin density of the Co active sites on the support surface, eventually optimizing the binding energy between surface Co and O species and promoting the OER activity.^86^

Although the number of studies covering the integration of multiple metal sites is rapidly increasing, reporting diverse electronic cooperative mechanisms between metal centers, general geometry-electronic property relations have not yet been unraveled. While the intersite distance has been proposed as a descriptor to quantitatively evaluate the electronic metal–metal interactions,^84,87,88^ rationalization of intermetallic catalytic synergies should not overlook other structural features, such as local coordination,^73,89^ for accurate analysis of orbital hybridization and charge transfer effects.

Finally, applying electromagnetic fields or controlled voltage may offer a promising strategy to tune the orbital configuration and spin state of the metal active sites.^90^ Future studies are encouraged to explore these aspects, holding promise for exciting discoveries.

## Characterization Approaches

### Established Methodologies

**Figure 4:**
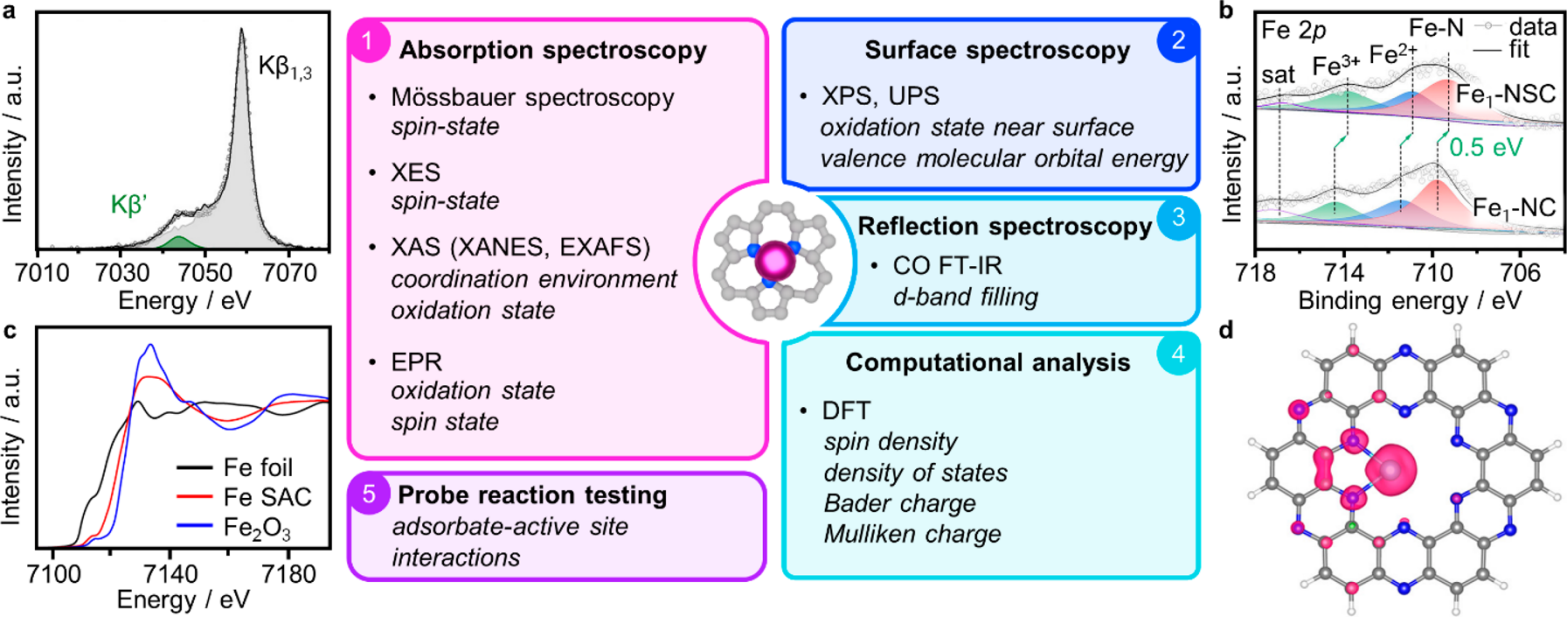
Experimental and computational approaches to characterize the electronic properties of SACs, accompanied by analysis results for carbon-supported Fe SACs from representative techniques. (a) Kβ mainline XES spectra, which derive from 3*p*–3*d* exchange interactions and whose spectral features depend on the Fe spin state. (b) Fe 2*p* XPS and (c) Fe K-edge XANES spectra, showing the positively charged nature of the Fe atoms. (d) Spin density analysis, performed by DFT simulations and illustrating that the spin is mainly located on Fe (central atom) and the neighboring N and C atoms (blue and gray atoms, respectively). [Panel (a) was adapted with permission from ref (103). Copyright 2021, John Wiley and Sons. Panel (b) was adapted with permission from ref (91). Copyright 2022, John Wiley and Sons. Panel (c) was adapted with permission from ref (92). Copyright 2021, Springer Nature. Panel (d) was adapted with permission from ref (94). Copyright 2021, American Chemical Society, Washington, DC.]

A standard approach for investigating the electronic features of SACs consists of assessing the formal oxidation state by XPS and X-ray absorption near edge structure (XANES), usually complemented by EXAFS to resolve the metal atom coordination environment (Figure 4).^4,91,92^ Aiming to gain atomistic insights into the active site structure, theoretical simulations often further integrate these experimental techniques by predicting the thermodynamic stability of metal species in different potential oxidation and coordination environments (Figure 4).^93^ Furthermore, computational studies typically aim to identify key electronic parameters for catalytic behavior rationalization by evaluation of adsorbate binding energies.^8,94^ However, SACs often present unique electronic features (vide supra), invalidating well-established catalytic descriptors (i.e., the *d*-band center) in extended metal surfaces. Unfolding the complex electronic structure of SACs and how the latter impacts the metal atom reactivity are nontrivial tasks that require a multitechnique approach. In this section, we comprehensively analyze advances and limitations in resolving key electronic properties, such as the band structure or spin configuration (vide supra), of characterization methods both experimental, consisting of absorption, surface, and reflection spectroscopic techniques, and computational (Figure 4). Thereafter, characterization approaches bridging the study of the active site electronic structure with its reactivity are discussed, examining recently developed approaches such as in situ and operando techniques, and highlighting future directions to unravel research areas that are often overlooked or vastly unexplored.

### Band Structure

A primary example of the unique, and complex, electronic structure of atomically engineered materials are SAAs. Although the catalytic properties of diluted metal atoms supported on a transition-metal matrix have been explored for a wide range of applications for almost two decades,^95^ their understanding in relation to the active site electronic structure still represents a major challenge to the heterogeneous catalysis research community. Only in 2018, computational analysis screening 15 different metal combinations revealed that many exhibited sharp *d*-band features near the Fermi level for the diluted metal atoms, pointing to such a feature as the cause of the remarkable reactivity.^15^ Following this study, pioneering work on AgCu alloys demonstrated the electronic isolation of diluted Cu atoms in the Ag matrix, introducing the concept of “free-atom-like electronic structure”.^16^ For this purpose, characterization by ultraviolet photoelectron spectroscopy (UPS), which is particularly useful for determining the valence electron structure of solid surfaces,^6^ was coupled with analysis of the PDOS by DFT. While UPS spectra exhibited a clear, sharp narrowing in the *d*-band of the Cu atoms when diluted in the Ag matrix, the PDOS showed that the Cu 3*d* line shape is nearly symmetric, indicating that the Cu 3*d* states do not strongly hybridize with the neighboring Ag atoms. When adsorbates hybridize with narrow valence bands (such as a *d*-band), the adsorbate state can split into localized bonding and antibonding states, resulting in an increased interaction strength with the catalyst surface.

Interestingly, analysis of the metal atom oxidation state by XPS, XANES, and DFT displayed charge transfer from the Ag matrix to the Cu single atoms, whereas EXAFS analysis of the metal bond length evidenced that the Ag–Cu bond in the SAAs is longer than the Cu–Cu bond in bulk copper, suggesting the presence of strain effects. Nevertheless, while the effect of band narrowness on catalytic performance was uncovered, the role played by metal atom-support charge transfer and metal–metal bond length remains unclear. Albeit both factors were shown not to affect the *d* bandwidth in the AgCu SAAs, later studies on other SAA systems reported their influence on the catalytic performance by enhancing reactive intermediate adsorption properties.^96,97^ In contrast, a recent theoretical study on SAAs demonstrated that critical shifts in the *d*-band center are associated with the formation of new electronic states in response to alloying, rather than with intermetallic charge redistribution.^98^ In the future, systematic studies investigating a range of metal–metal combinations are encouraged to unambiguously establish descriptors for the SAA electronic structure of SAAs, requiring mandatory validation by theoretical models.

To date, computational efforts have been mainly directed to rationalizing and predicting metal site–adsorbate binding mechanisms. These encompassed the study of frontier molecular orbitals, highest and lowest unoccupied molecular orbitals of both adsorbates and single metal sites,^50,51^ as well as Bayesian learning of chemisorption processes.^99^ A recent DFT study examining the use of transition-metal-based SACs for the ORR found that the activity of SACs with partially filled *p* orbitals is controlled by the position of the *p*-orbital energy levels relative to the Fermi level. This suggests that the electronic properties of the *p* orbitals, rather than just the *d* orbitals, play a crucial role in determining the catalytic activity of these SACs.^100^

Although all the above-mentioned approaches are valuable for understanding SAC reactivity, they still require expensive and complex simulations that are not easily accessible to researchers attempting to design efficient SACs for a specific reaction. Lately, an uncommonly simple molecular orbital approach has been proposed for SAAs based on the key concept that the interaction of adsorbates at their surface can be described in terms of an electron counting rule.^17^ By combining machine learning and DFT analyses, a variety of catalytically relevant adsorbates was screened on a large set of SAA surfaces, showing that the adsorbate interaction with the isolated metal site is maximized when the *n*_*d*_ valence electrons of the dopant and the *k* valence electrons of the adsorbates sum up to 10: *n*_*d*_ + *k* = 10. Undoubtedly, this simple 10-electron rule provides experimentalists with a straightforward tool to identify suitable metal combinations when designing SAAs for targeted applications. Nonetheless, one should be mindful that the rule applies to systems that do not exhibit strong electrostatic interactions between the adsorbate and the SAA surface nor that feature 3*d* isolated metal atoms.

### Spin Configuration

Because of their magnetic nature, the reactivity rationalization of SACs featuring 3*d* metal single sites requires thorough investigation of the active site oxidation and spin state, as well as investigation of its spin polarization (i.e., magnetic moment).^51,101^ While these descriptors can be evaluated by computational DFT methods, including PDOS and Wannier function analyses, their assessment by experimental characterization techniques is often hindered by the latter’s intrinsic limitations. For example, Mössbauer spectroscopy and electron paramagnetic resonance (EPR) spectroscopy can provide valuable insights into the spin polarization configuration (Figure 4).^71,102^ However, the former can only be applied to Mössbauer-active transition metals while the latter can probe only paramagnetic sites. Furthermore, the long acquisition times for Mössbauer spectra do not render the technique suitable to study dynamic effects under reaction conditions. To overcome all these limitations, a novel, nonresonant approach based on X-ray emission spectroscopy (XES) has been proposed for the quantification of the metal site average spin state (Figure 4).^103^ By virtue of the short acquisition times of in situ experiments over a few minutes, a recent XES study on a carbon-supported Fe SAC for the electrochemical oxygen reduction reaction (ORR) provided experimental proof of potential-induced changes in the Fe SAC spin state. Although the number of studies on 3*d* metal-based SACs has been increasing over the past few years, especially on Fe SACs, our understanding of their electronic and catalytic properties remains inadequate to derive general structure-performance relations. For this purpose, it will be pivotal to develop spin configuration characterization techniques that not only are applicable to all metals and under operando conditions, but also enable one to gain insights into features, such as the magnetic moment, that are currently accessible only via computational analyses.

In stark contrast, many experimental techniques are available to assess the electron density distribution in metal sites upon interaction with reactive molecules. For example, in situ Fourier-transform infrared (FT-IR) spectroscopy of CO chemisorption at room temperature was shown to be a convenient tool to disclose the population degree of *d*-state electrons in Ru SACs, supported on Ni- and Fe-based layered double hydroxides, for the benzyl alcohol oxidation reaction.^104^ Nevertheless, probe molecules themselves can trigger changes to the SAC surface and active sites, offsetting the insights gained through IR characterization.^105^ Although preventive measures can be taken by conducting the IR analysis under cryogenic conditions, often quenching active site restructuring, it is recommended to resort to other complementary characterization techniques that do not require the use of probe molecules.

### In Situ and Operando Techniques

Ambient pressure XPS has recently emerged for in situ and operando studies of the oxidation state of surface metal sites in SACs.^106−109^ However, it still suffers from limited resolution for highly diluted elements and scarce applicability to liquid-phase reactions.^109^ In contrast, XAS, including XANES and EXAFS, has been successfully employed to determine the metal site oxidation and coordination environment under various reactive atmospheres. Over the past decade, an ever-increasing number of in situ and operando XAS studies has been performed on SACs in a variety of applications, encompassing thermochemical, electrochemical, and photochemical reactions.^4,110−113^ This has propelled the development and use of advanced techniques, including (i) high-energy-resolution fluorescence detection XANES, enabling the resolution of the metal site electronic structure even in the most dilute samples under reactive atmospheres,^114,115^ (ii) combined operando XAS and X-ray powder diffraction (XRD) analyses,^116,117^ elucidating mechanisms for oxidation state and crystallographic structural changes in the support that prompt metal active site restructuring, as well as (iii) processing methods for the large volumes of data acquired in operando investigations.^118^

These remarkable advances pave the way for synergistic approaches combining operando spectroscopic analyses with computational simulations, rationalizing dynamic charge redistribution effects under reactive environments,^119^ and ultimately uncovering the active site electronic structure and reaction mechanism. To this end, experimentalists should attempt to obtain a better overview of the dynamic processes occurring in the catalytic material. To date, there is a lack of operando XAS studies probing at the same time the diverse elements present in the SAC under examination, which would unlock the investigation not only of metal–adsorbate and metal–support interactions, but also of adsorbate–support ones. This undertaking should be further complemented by in situ and operando soft XAS analyses. These are sensitive to investigating the bond types and orbital hybridization of light elements such as C, N, O, S, and Cl commonly encountered in carbon-based hosts and exchangeable ligands.^109^ Furthermore, given research interests shifting toward more complex, multimetal SACs,^120^ multielement operando XAS studies will be highly valuable for deriving robust structure–performance relations.

### Future Directions

While advanced synchrotron-based X-ray absorption and diffraction analyses offer rich information on the SAC oxidation and coordination environments, basic electrochemical techniques such as cyclic voltammetry, electrochemical impedance spectroscopy, and Tafel analyses can provide valuable insights, e.g., into the rate of electron transfer.^6^ Another often overlooked experimental approach to gain insights into the properties of active sites is probe reaction testing, as the adsorption strength of reactive intermediates strongly depends on the metal atom electronic structure.^121−123^ Additionally, when combined with well-defined reference materials and further complemented by operando spectroscopic techniques, probe reaction testing targeting metal site exposure to selected atmospheres can enable the study of the dynamic evolution of the oxidation and coordination environments under conditions that are relevant to a wide range of applications.

Finally, we highlight that, while the overwhelming majority of current studies intend to derive electronic–catalytic property relations on a case-by-case basis, future efforts should be devoted to identifying general electronic descriptors applicable to SACs, regardless of their composition, metal site architecture, and catalytic applications. Furthermore, research studies should not overlook catalytic material heterogeneity and extensively employ methodologies characterizing the uniformity of metal species, to reliably resolve the active site structure.^124−126^ Because of the sensitivity of individual techniques to specific catalyst properties and their limitations,^127^ extensive systematic investigations should be conducted employing holistic approaches that combine multiple experimental and computational techniques. This will be essential to comprehensively probing all key electronic characteristics of SACs (Figure 1) and obtaining a complete understanding of electronic–catalytic property relations.

## Impacts on Reactivity

**Figure 5:**
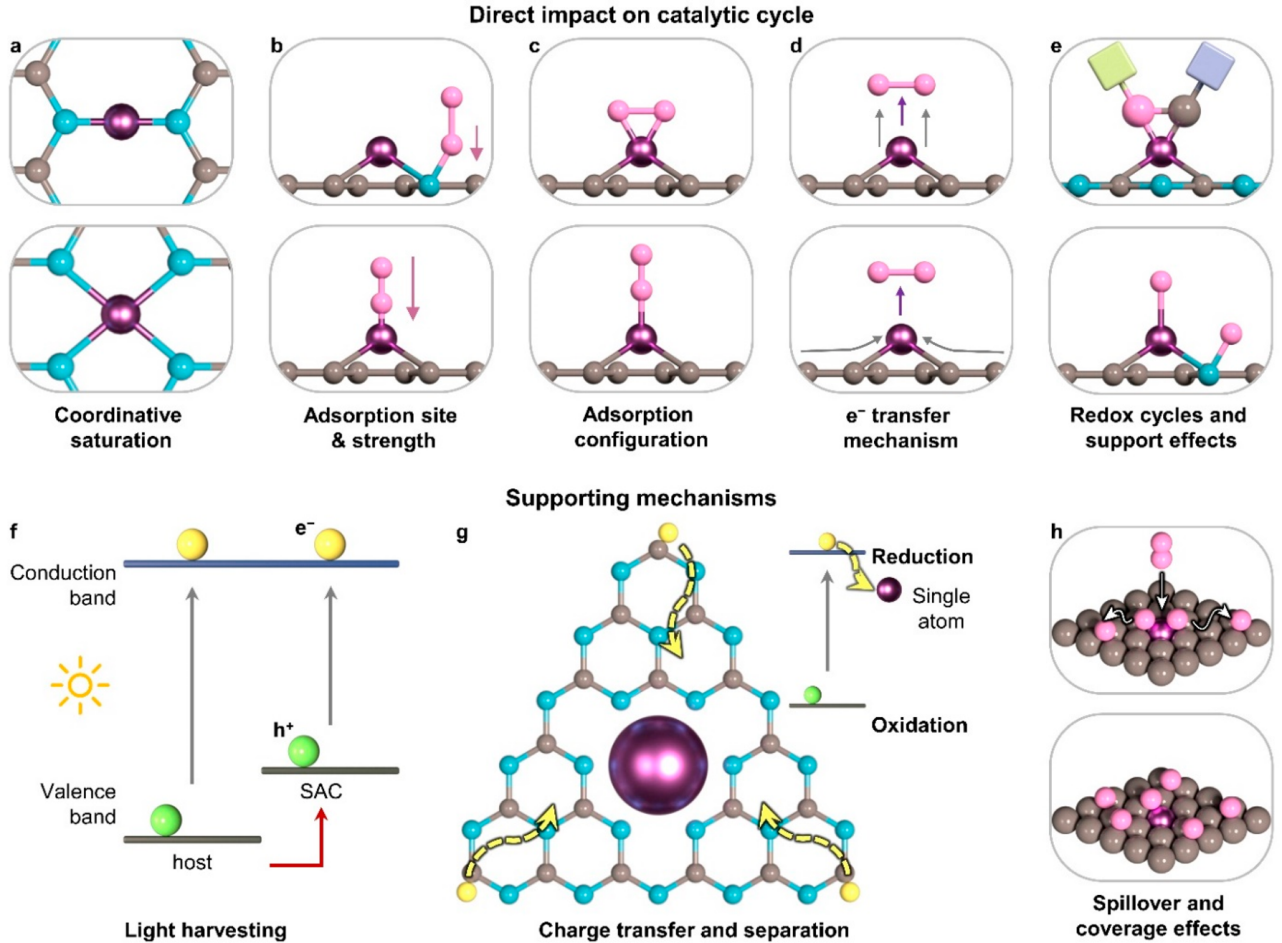
Schemes illustrating the potential effects of the electronic structure of single-atom catalysts on their performance. These impacts can include both direct influences on the catalytic cycle and surface catalytic reactions (a–e) and indirect effects on supporting mechanisms (f–h). The electronic properties of the support can also play a role in both direct and supporting mechanisms. By carefully controlling these properties, it is possible to enhance catalytic efficiency and ensure stable performance.

Optimizing the electronic structure of SACs to enhance their reactivity in targeted applications requires analysis of how they adsorb and activate reactant molecules, break and form specific bonds within or, given sufficient proximity, between intermediates species, and desorb products. Strategies may focus on directly optimizing the desired catalytic cycle or indirectly improving the efficiency of supporting mechanisms (see Figure 5). A critical challenge is catalyst stability, which depends strongly on the electronic structure of metal centers. The support can play a pivotal and sometimes overlooked role in all cases. Here, we review the understanding of each of these effects.

### Catalytic Cycle

The ability of metal atoms to chemisorb reactants relies on their coordinative unsaturation. A common misconception encountered in the SACs literature is that the dispersion of metal nanoparticles into single atoms will automatically result in the ultimate fraction of coordinatively unsaturated species following the behavior typically associated with decreasing particle size. However, the coordinative unsaturation of metal centers in SACs cannot be assumed, and several examples have emerged that reveal a strong dependence on the SAC composition and synthesis method (Figure 5a). For example, elevated temperature treatments to remove ligands can induce strong coordination with supports resulting in limited activity, as reported for Pt supported on nitrogen-doped carbons for acetylene hydrochlorination and Pt on CeO_2_ for CO oxidation.^128,129^ A challenge driving ongoing research is how to design SACs with optimized valence states, which may be low or high, depending on the application, while preserving high stability, e.g., against sintering or leaching (vide infra).^130^ In the case of Pt_1_/CeO_2_, the coordinative saturation of Pt atoms was linked to that oxidation state of the support, in particular by the number of Ce^3+^ centers formed via electron transfer into the 4*f* orbitals of Ce^4+^.^131^ Lower coordination to supports can potentially be induced by the adsorption of reaction components if they create a stable environment for the metal.^11,50^

**Figure 6:**
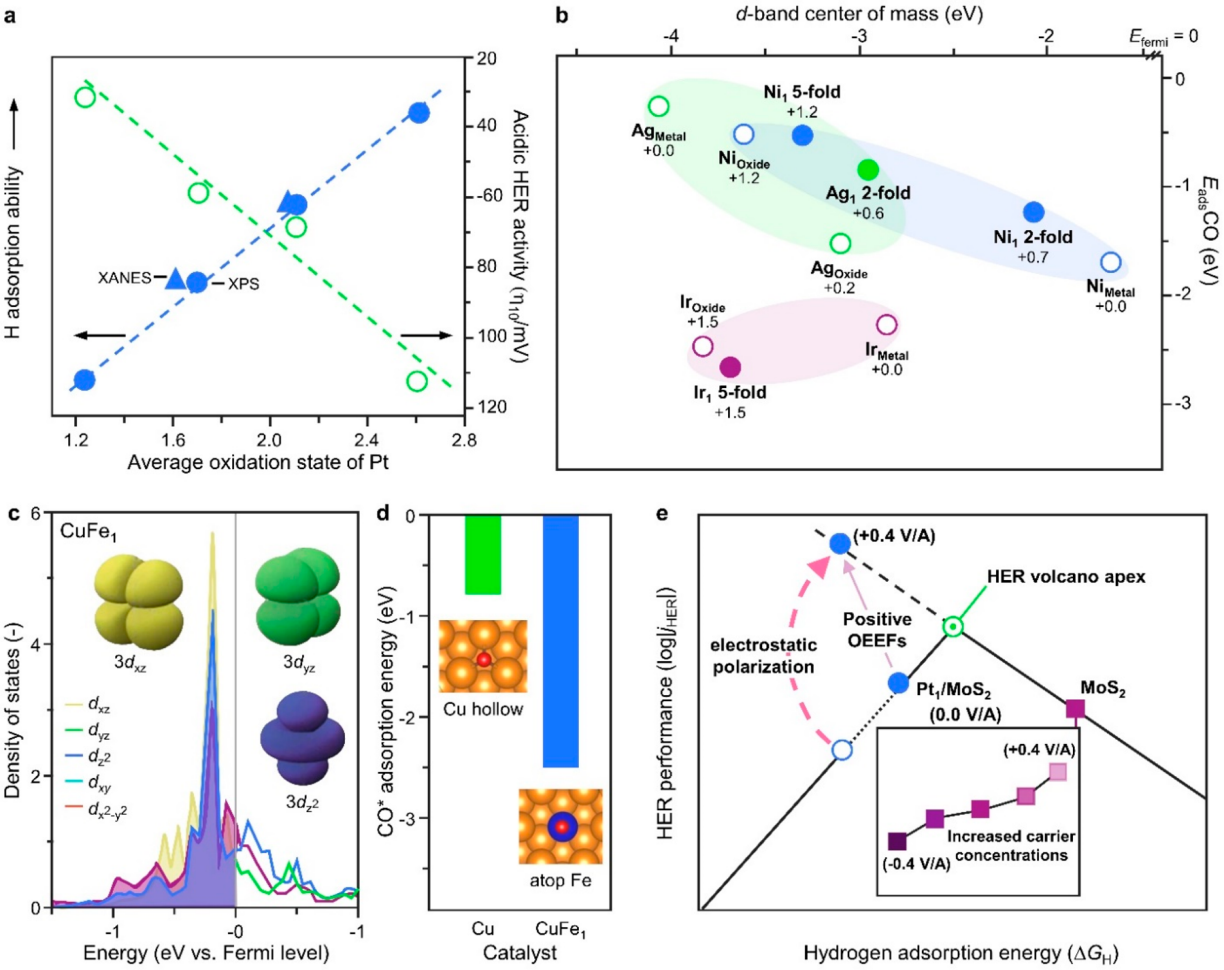
Examples exploring the relationship between SAC electronic structures and performance. (a) Relationship between the average oxidation state (determined by XPS or XANES), H adsorption ability (quantified by the position of the *d*-band center), and acidic HER activity of transition-metal dichalcogenide-based Pt-SACs. (b) CO adsorption energy for M_1_/Fe_3_O_4_(001) SACs compared with the metal (M_Metal_) and metal oxide (M_Oxide_) surfaces, plotted against the *d*-band center of mass. (c) Deconvolution of the density of states of the *d*-orbitals of a single Fe atom in Cu–Fe SAA (CuFe_1_) and (d) the adsorption energy of *CO on the Cu–Fe SAA compared to pristine Cu. (e) Positive oriented external electric field (OEEF)-induced breakthrough at HER volcano apex (*j*_HER_ – Δ*G*_H_) in MoS_2_-based Pt SACs. The inset shows the HER performance of pristine MoS_2_ associated with the OEEF-regulated carrier concentration. [Panel (a) was adapted with permission from ref (132). Copyright 2021, Springer Nature. Panel (b) was adapted with permission from ref (11). Copyright 2021, AAAS. Panels (c) and (d) were adapted with permission from ref (57). Copyright 2022, Springer Nature. Panel (e) was adapted with permission from ref (90). Copyright 2022, Springer Nature.]

As a readily experimentally determinable parameter, changes in valence states resulting from charge transfer to neighboring atoms have been reported to influence the strength, location, configuration, and electron transfer mechanisms of molecular adsorption (Figures 5b–d). Several direct correlations have been identified between metal oxidation state and activity, although these have typically been observed in studies of SACs based on the same metal on different supports. For example, the comparison of Pt SACs based on transition-metal dichalcogenides (MoS_2_, WS_2_, MoSe_2_, and WSe_2_) correlated the acidic/alkaline HER activity with the average oxidation state of Pt single atoms, determined by XPS or XANES, and the H or OH adsorption ability, evaluated through analysis of the *d*-band center (Figure 6a).^132^ In selective hydrogenations, DFT simulations of Pt_1_/Fe_2_O_3_ catalysts linked increasing adsorption strengths of H_2_ to decreasing oxidation states, varied by controlling the Pt–O coordination number.^121^ In the case of Pd SACs based on different crystalline forms of carbon nitride, they further showed that the mechanism of hydrogen activation could follow different paths (homolytic or heterolytic), depending on the electronic structure.^133^ In the heterolytic case, the proton is adsorbed on a neighboring N-group in the support. Distinct relationships with oxidation state have been reported in other applications. For example, a volcano dependence was observed for Os-based SACs in the HER, linked to the adsorption strength of H atoms.^134^ A descriptor combining the valence electron number of metal, electronegativity of central metals, and radius of the metal ions was also proposed for the activity in the electrocatalytic CO_2_ reduction reaction (eCO_2_rr), evidencing volcano-like correlation for SACs based on a range of different transition metals,^135^ which was subsequently linked to a direct influence on the bonding strength with intermediates.^136^

Despite its broad applicability for describing the reactivity of supported transition-metal nanoparticles, the *d*-band model is generally not suited for SACs, because of the chemical bonding that typically occurs between isolated metal centers and their supports.^11^ Nonetheless, quantum chemical calculations have demonstrated the importance of considering properties of the *d*-band structure as descriptors for adsorption, and they have highlighted the intimate dependence on the local atomic environment.^137^ The complexity of linking the electronic structure of SACs to the strength of reactant adsorption was shown by comparing single atoms of different metals (Cu, Ag, Au, Ni, Pd, Pt, Rh, Ir) occupying the same 2-fold coordination of a model Fe_3_O_4_ support (Figure 6b).^11^ Charge transfer to the support could strengthen or weaken the metal–CO bond, but CO-induced structural distortions reduce adsorption energies from those expected based on the electronic structure alone, illustrating the limited applicability of the *d*-band center of mass as a predictor of CO adsorption energies in SACs.

The ability to control the configuration of adsorbed species presents exciting opportunities for directing the selectivity of reactions. By using a copper-supported iron SAC, it was possible to catalyze the hydrogenation of CO_2_ to methane, where single atoms stabilized on nitrogen-doped carbon typically only could achieve the conversion to CO due to the weak adsorption of CO intermediates.^57^ Analysis of the density of states of the *d*-orbitals showed that the unique selectivity likely originated from the mainly *z*-axis character (Figure 6c), resulting in the preferred adsorption of *CO on atop sites of the Fe atoms. The binding of *CO was stronger in this geometry compared to at the hollow sites of the neighboring Cu surface (Figure 6d), promoting the preferential transfer to the top Fe locations. In catalyzed reactions involving redox cycles, the capability of adaptive coordination to the support is an indispensable design feature, enabling metal centers to supply or take up electrons to satisfy the mechanistic requirements (Figure 5e).^138,139^ This property is thought to be favored by structurally flexible supports with polydentate coordination sites, where the metal center can access different coordination states of similar energy. This has also been linked to ensuring the effective stabilization of single atoms during challenging reactions, where the coordination to the support may be significantly reduced during the catalytic cycle. The redox-tunability of supports can also play a critical role, as shown for a Rh SAC for CO oxidation, where the reoxidation of a heteropoly acid support was found to be rate-limiting.^106^

Besides precisely controlling the nanostructure of SACs, regulation of the electronic properties may also be achieved by applying external fields during the catalytic reaction. Superior electrocatalytic performance was reported in SACs under electrostatic modulations, by oriented external electric fields (see Figure 6e).^90^ Simulations suggest that the electrostatic fields boost the performance by significantly polarizing the charge distributions at the single-atom sites and alter the kinetics of the rate-determining step. Alternating magnetic fields were also proposed as a promising methodology to improve the activity of a MoS_2_-supported cobalt SAC in the OER.^140^ These very recent studies shed light on innovative approaches for further development of SACs for sustainable chemical transformations. An aspect that is often overlooked is that support materials may also directly participate in fulfilling the catalytic cycle (Figure 5e). In addition to alkyne hydrogenation, the metal and the support were both reported to participate in C–C coupling over Pd atoms supported on TiO_2_.^141^ Identifying supports that can provide complementary properties will likely be a valuable strategy for overcoming the limitations of SACs in catalyzing more complex reactions that require the adsorption of more than one species, where otherwise they would be inactive.^142^

### Supporting Mechanisms

The electronic properties of SACs can also benefit supporting mechanisms for the catalytic cycle that boost the performance. As supports influence the electronic structure of metal centers anchored on them, the atomic dispersion of metals can also impact the band structures of carrier materials, which can affect their light absorption behavior and charge transfer properties (Figures 5g and 5h).^143−146^ Recently, more dedicated studies have emerged aimed at improving fundamental understanding of these effects. DFT simulations of graphitic carbon nitride doped with Pd, Pt, and Au evidenced significantly narrower band gaps compared to the metal-free support, that could extend the light absorption range and hence utilization ability in photocatalytic applications.^147^ This possibility provides unique opportunities to tune the band-edge energy levels of supports to meet the requirements for specific redox reactions by promoting charge transfer to enable a sufficient driving force.^145^ For example, by downshifting the valence band by 0.26 V, compared to the metal-free support, the introduction of single Pt atoms in graphitic carbon nitride was shown to enable direct photocatalytic water splitting in pure water.^148^

The unique metal–support interactions can also benefit charge-transfer processes in photocatalytic and electrocatalytic systems (Figure 5g).^143,144,146^ The possibility to integrate active metal centers into light harvesting materials shortens the distances required for the transfer of photogenerated electrons to drive catalytic processes, generally enhancing the efficiency by minimizing the possibility of recombination with the carrier or relaxation during charge migration.^149−151^ Besides reducing the length of charge transfer paths, several works have demonstrated the possibility for atomically dispersed metals to trap photoexcited electrons, promoting charge separation and localization on the active centers.^152^ Studies of SACs of different metals based on metal–organic frameworks have determined that the ability depends on the chemical identity of the metal.^149−151,153^ In addition, it has been shown that the presence of metal atoms can facilitate charge by migration across the interlayer of 2D lattices by acting as electron transfer channels, as demonstrated for palladium SACs based on graphitic carbon nitride.^154^

Finally, the electronic structure of the support can also influence other established mechanisms such as hydrogen spillover and coverage effects (Figure 5h), although this is not widely studied. Research on materials with specific nanostructures found that hydrogen spillover from single Pd atoms to a copper support can depend on the surface facet.^155^ In the context of sustainable catalysts for acetylene hydrochlorination, carbon supports are known to act as acetylene reservoirs but the impact on the performance remains unclear.^156^ As more research is conducted, additional examples of these effects may be discovered.

### Catalyst Stability

As highlighted, an important relationship exists between the interaction of metal atoms with supports, their electronic structure, and their stability in catalytic applications (Figure 5). Both the energy supplied to drive the reaction and the interaction with reactive intermediates, solvents, or electrolytes influence the geometric and electronic structure of the active site.^127^ These structural changes can destabilize chemical bonding of single atoms with supports, increasing the tendency for SACs to deactivate through well-known mechanisms of sintering, leaching, or volatilization. Besides, the electronic properties of the SACs may result in overly strong binding of reactants, products, or other compounds present in the reaction mixture,^157^ which can cause catalyst deactivation due to poisoning or promoter depletion over time on stream or upon catalyst recovery and reuse.

Several strategies have been developed to preserve the active site structure and the metal oxidation state under reactive environments, namely by engineering the support or ligands and regulating the reactive environment.^130,158−163^ For example, to avoid diffusion and sintering of metal atoms on hosts that can poorly stabilize them, because of limited free chemical valence at anchoring sites and low barriers to migration, such as graphene,^158^ the substitution of C atoms with nonmetals has been widely pursued. The introduction of heteroatoms enhances the stability of the metal sites by the remaining free valence of undercoordinated C atoms at the defect site, heightening the sintering barrier.^164^ The choice of support was also shown to be critical to avoid leaching or poisoning by boron in the design of Pt catalysts for the dehydrogenation of ammonia borane.^130^ A magnetic Co_3_O_4_ support permitted a stable performance, which was attributed to a remarkable electronic perturbation induced by the magnetic support through strong EMSI, increasing the binding energy of single Pt atoms on Co_3_O_4_ compared to ZrO_2_ or graphene alternatives.

While host engineering provides a key strategy for enhancing the stability of SACs, potentially detrimental impacts must be considered.^159^ For example, the CO-induced coalescence of Pt atoms supported on a reducible Fe_3_O_4_(001) surface at room temperature was linked to the cleavage of metal and O atoms in the lattice of the support upon adsorption of reductive gases (CO and H_2_) on the metal site.^160^ This change of the support structure induced a progressive shift in the Pt charge from positive to neutral, and the resulting Pt^0^ species aggregated into nanoparticles. Note that redox phenomena might dynamically occur during catalytic processes without leading to catalyst deactivation. Simulations on reducible oxide-supported Au nanoparticles for CO oxidation revealed that, upon CO adsorption, Au cations form and migrate onto the support to participate in the catalytic cycle before reintegrating back into the nanoparticles after completing the reaction.^161^

Other detrimental effects due to support modifications reported in different applications likely also result from a suboptimal electronic structure. For example, heteroatom modifiers introduced in carbons to enhance the metal–support interaction strength were linked to coke deposition in acetylene hydrochlorination.^122^ Similarly, the protonation of N-functionalities and subsequent deactivation upon complexation with counterions in the electrolyte is a prominent cause of catalyst degradation in the ORR reaction over Fe SACs based on N-doped carbons, requiring the latter’s removal by thermal or chemical treatment for regeneration of the active site.^165^ However, the molecular-level origin of these effects is not always fully understood. It is noteworthy that the anchoring of metal atoms may also impact the support properties, having positive or negative effects without involving other modifiers.^166−170^

Finally, alternative approaches encompassing ligand engineering and regulation of the reactive environment can also effectively modulate the electronic properties, enhancing the stability of SACs. For example, irreversible and detrimental redox phenomena were successfully hampered for Ni centers catalyzing the eCO_2_RR by integrating electron-donating methoxy groups in the protective phthalocyanine ligands.^162^ Similarly, the application of ionic liquids as reaction solvents can enable regulation of the electron distribution at the active site through charge transfer between anions and cations with both the metal sites and the support. This can ultimately lead to higher binding energy and resistance to leaching of the single atoms, as recently reported for TiO_2_-supported Rh SACs for hydroformylation reactions.^163^

In summary, the modulation of SAC electronic features is a complex undertaking that can enhance catalytic activity and selectivity and unlock stable performance. Still, the development of effective strategies requires detailed knowledge of the role of the material’s electronic properties in fulfilling the catalytic cycle. To this end, future studies are encouraged to adopt holistic approaches investigating the active phase in its integrity, encompassing metal sites, ligands, and supports, as well as their potential interactions with the reaction media.

## Conclusions and Outlook

Unlocking the full potential of single-atom catalysts requires precise knowledge of how to tailor their electronic structure and associated stability, conductivity, optical, and magnetic properties. This Perspective has highlighted recent developments in understanding how the electronic characteristics of SACs can differ from those of supported metal nanoparticles, synthetic and analytical approaches for modulating their properties, and the potential catalytic impacts reported. Although various procedures are known for tuning the electronic properties, these have often been proposed to explain observed behavior rather than applied as design strategies. Attaining complete control over the electronic structure of SACs remains nontrivial. Currently interpretations are often based on simulations making certain structural assumptions, for example, supposing regular active site structures and neglecting potential dynamic effects. Furthermore, the simulations often rely on information derived from the ex situ analysis of SACs, which may not reflect the behavior of practical catalysts under reaction conditions. Despite these limitations, simulations play a valuable role in guiding us to the potential range of accessible electronic property variations as a function of distinct structural features.

Overcoming current synthetic and analytical frontiers will bring tremendous progress in the atomically precise design of catalytic materials. The analysis undertaken in this Perspective identified several priorities for moving forward. It emphasized the need for more dedicated efforts exploring the design of supports with well-defined coordination site architectures that provide optimal environments to maximize the catalytic efficiency of anchored metal atoms. Desirable features include the possibility to regulate orbital occupancy, band edge energy levels, and spin states, providing multidentate structures that permit adaptive coordination during the catalytic cycle, and harnessing synergies with the support to enhance charge transfer and light absorption properties. Alongside developing SACs with better defined structures, advances in smart characterization approaches seamlessly integrating computational modeling and experimental analysis into efficient workflows guided by artificial intelligence can accelerate and improve the accuracy of discriminating the electronic structure of SACs through commonly applied techniques such as X-ray absorption, X-ray photoelectron, and electron paramagnetic resonance spectroscopy.
